# Electrospinning in promoting chronic wound healing: materials, process, and applications

**DOI:** 10.3389/fbioe.2025.1550553

**Published:** 2025-03-06

**Authors:** Jiaxi Kou, Yaodong Li, Chen Zhou, Xiyu Wang, Jian Ni, Yue Lin, Huaqiang Ge, Dongfeng Zheng, Guopu Chen, Xitai Sun, Qian Tan

**Affiliations:** ^1^ Department of Burns and Plastic Surgery, Nanjing Drum Tower Hospital Clinical College of Nanjing Medical University, Nanjing, China; ^2^ Department of Pancreatic and Metabolic Surgery, Nanjing Drum Tower Hospital, Affiliated Hospital of Medical School, Nanjing University, Nanjing, China; ^3^ Department of Pancreatic and Metabolic Surgery, Medical School of Southeast University, Nanjing Drum Tower Hospital, Nanjing, China

**Keywords:** electrospinning, hydrogel, nanofiber, wound healing, dressing

## Abstract

In the field of wound treatment, chronic wounds pose a significant burden on the medical system, affecting millions of patients annually. Current treatment methods often fall short in promoting effective wound healing, highlighting the need for innovative approaches. Electrospinning, a technique that has garnered increasing attention in recent years, shows promise in wound care due to its unique characteristics and advantages. Recent studies have explored the use of electrospun nanofibers in wound healing, demonstrating their efficacy in promoting cell growth and tissue regeneration. Researchers have investigated various materials for electrospinning, including polymers, ceramics, carbon nanotubes (CNTs), and metals. Hydrogel, as a biomaterial that has been widely studied in recent years, has the characteristics of a cell matrix. When combined with electrospinning, it can be used to develop wound dressings with multiple functions. This article is a review of the application of electrospinning technology in the field of wound treatment. It introduces the current research status in the areas of wound pathophysiology, electrospinning preparation technology, and dressing development, hoping to provide references and directions for future research.

## 1 Introduction

Wounds are formed when the integrity of the skin is compromised. If the wounds cannot heal for a long time, they will evolve into chronic wounds (Falanga et al., 2022). This has brought a considerable burden to the medical system. The highest wound-related costs are surgical incisions, followed by diabetic foot ulcers (Graves et al., 2022). There are millions of patients suffering from chronic wounds every year. Prevention of wound infection, correct care, and appropriate dressing are important factors in promoting wound healing (Las Heras et al., 2020). Nowadays, many mature wound dressings have been manufactured, such as gauze (Montaser et al., 2020), films, microneedles (Chi et al., 2020), hydrogel (Chen G. et al., 2023; Huang J. et al., 2023), and foams (Montazerian et al., 2022). The ideal dressing should possess good breathability and biocompatibility while exhibiting low cytotoxicity. Despite the development of various types of dressings, most face challenges such as high manufacturing costs and difficulties in large-scale production. Bacterial colonization in wounds forms biofilms, significantly impacting healing rates (Darvishi et al., 2022). Addressing biofilm removal presents an additional challenge in dressing development.

Electrospinning, first reported in 1934, is a continuous process for producing nanofibers with smaller diameters than mechanical drawing, gaining widespread study in the 1990s (Li and Xia, 2004a). Electrospinning has a large surface area, porous structure, and good biocompatibility (Xue et al., 2019). By changing the modifiers, different chemical properties can be obtained. In the early 21st century, electrospinning began to be widely used in the development of wound dressings (Rho et al., 2006; Ignatova et al., 2007). So far, a variety of wound dressings prepared using natural or polymer electrospinning have been developed (Castro et al., 2021; Wang et al., 2021). Electrospinning technology can be used to make fiber membranes with various functions such as antibacterial, breathable, and pH adjustment (Chen et al., 2022). Electrospun fibers that mimic extracellular matrix have good air permeability, high porosity, and considerable drug loading capacity, providing an excellent environment for tissue regeneration. It has great potential for application in wound treatment. Chitosan-based nanofibers blended with other polymers have been widely used in the biomedical and pharmaceutical fields (Augustine et al., 2020). Electrospun polycaprolactone (PCL) fibers embedded with two-dimensional nanovermiculite can promote angiogenesis and collagen deposition in diabetic foot ulcers, thereby accelerating wound healing (Huang X. et al., 2022). In addition to facilitating the healing process, electrospun fibers, when used as wound dressings, can also reduce wound tension, thereby minimizing skin scar formation (Mulholland, 2020).

**SCHEME 1:**
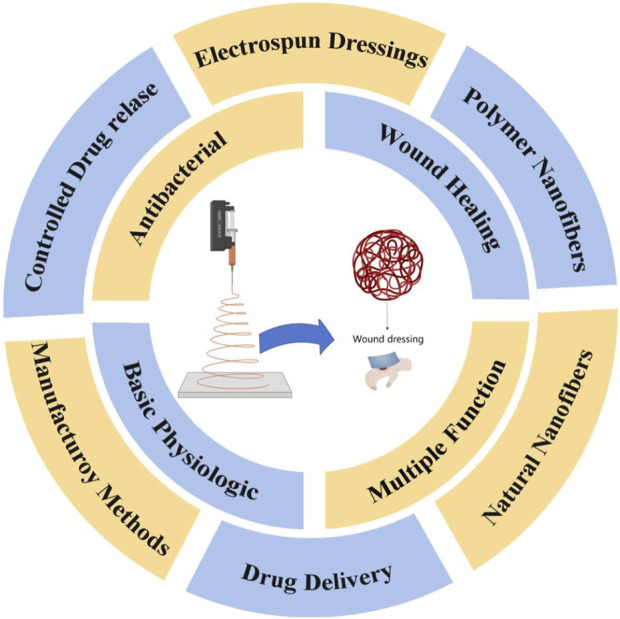
Electrospun fibers prepared from polymers or natural raw materials offer advantages such as excellent breathability, high biocompatibility, and ease of fabrication. Additionally, they possess antibacterial properties, controlled drug release capabilities, and drug delivery functions, making them highly promising for wound treatment applications.

This article introduces the fundamental principles and production process of electrospinning. Herein, electrospinning is classified from the aspects of raw materials, structure, and preparation process. Their application status in wound dressings is also provided. Finally, this article analyzes the shortcomings and challenges of electrospinning in wound dressings, and looks forward to future development prospects to provide reference for future research (Scheme 1).

### 1.1 Basic physiologic response to wound healing

**FIGURE 1:**
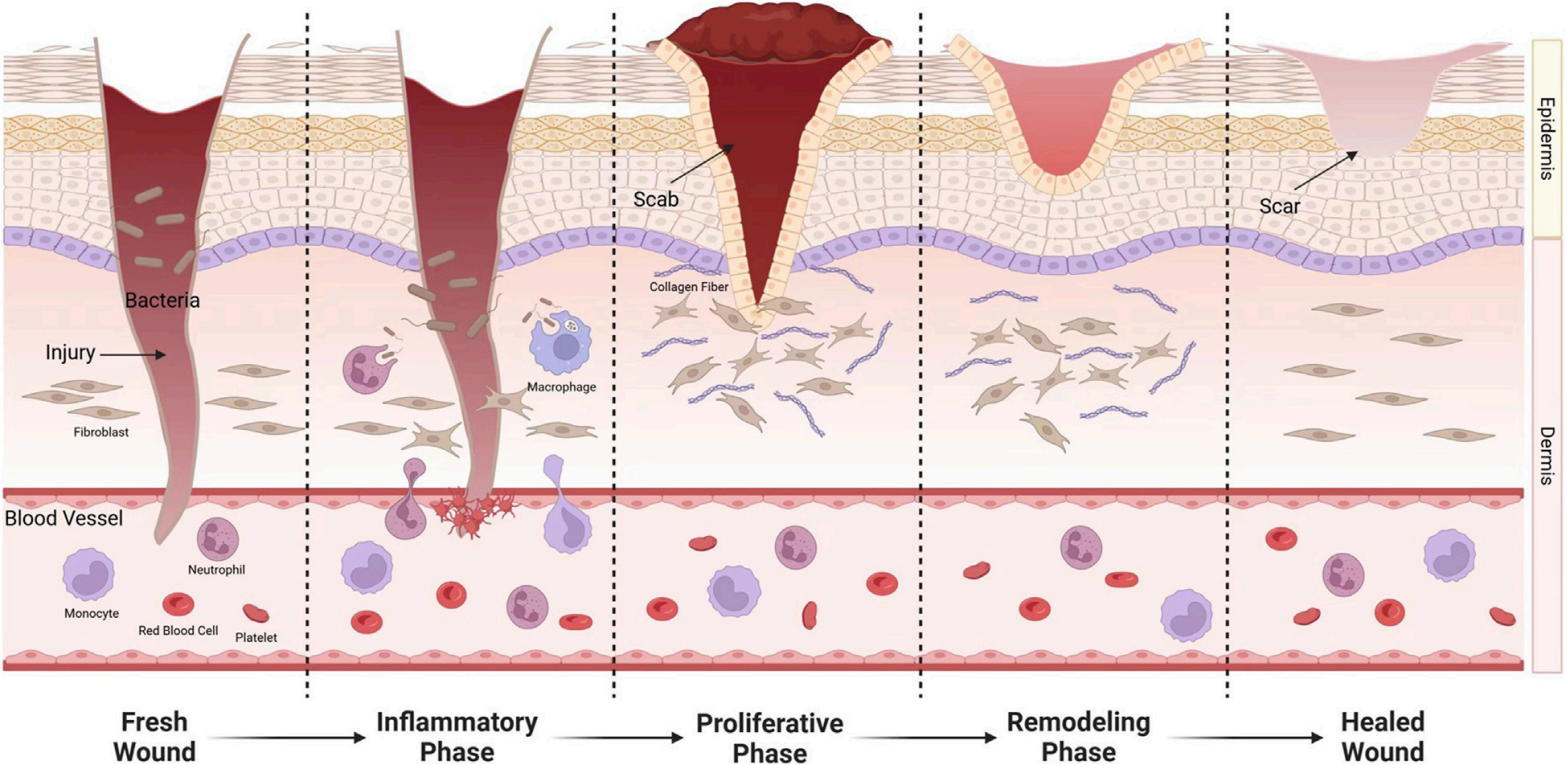
After injury, exposure of endothelial cells causes platelet aggregation, adhesion, and activation. Fibrinogen is converted into fibrin to stop bleeding. Leukocyte chemokines released by platelets induce neutrophils, lymphocytes, and monocytes to the wound. Monocytes transform into macrophages. They not only remove invading microorganisms and necrotic tissue from the wound, but also release a variety of cytokines to promote wound healing. During the repair phase, fibroblasts enter the wound and synthesize proteins, some of which are converted into myofibroblasts.

The wound healing process has three main phases: inflammatory, proliferation, and remodeling. Obstacles at any of these stages may lead to chronic wounds. The first thing that occurs after an injury is the inflammatory phase. After a brief period of vasoconstriction, the vasculature and the release of growth factors and vasoactive substances such as PDGF, TGF-β, thromboxane, and histamine (Chang and Nguyen, 2021), thereby promoting chemotaxis of neutrophils and macrophages, leading to the onset of the inflammatory phase (Rodrigues et al., 2019). Platelet-derived growth factor (PDGF) is secreted by degranulated platelets and plays a crucial role throughout all stages of wound healing. During this process, PDGF initiates wound repair by chemotactically attracting neutrophils, macrophages, fibroblasts, and smooth muscle cells to the wound site, while also promoting cell proliferation through mitosis to enhance their numbers (Zarei and Soleimaninejad, 2018). Macrophages are critical to this stage, assisting in phagocytosis and promoting the release of cytokines and fibroblast proliferation (Caputa et al., 2019). Bacterial colonization of the site during this stage may result in an extended inflammatory phase, complicating the healing process (Hurlow and Bowler, 2022; Eming et al., 2017). The period lasts for about 48 h, after which fibroblasts become predominant in the wound healing process (Shook et al., 2018). During the repair phase, fibroblasts play the key role. Fibroblasts produce matrix metalloproteinases that degrade fibrin clots and replace them with extracellular matrix (ECM). ECM regulates fibroblast activity and produces signals that influence angiogenesis, granulation tissue formation, and epithelialization (Bainbridge, 2013). New capillaries are generated and grow into the wound, providing substances necessary for wound healing (Stadelmann et al., 1998). This period is influenced by TGF-β1,PDGF, VEGF, angiotensin II, IL-1 and many other growth factors and cytokines (Barrientos et al., 2008; Rousselle et al., 2019). Vascular endothelial growth factor (VEGF) promotes wound healing by facilitating early angiogenesis and endothelial cell migration. It enhances blood flow to ischemic wound sites, thereby alleviating ischemia and hypoxia. At this stage, PDGF primarily functions to upregulate insulin-like growth factor-1 (IGF-1) and thrombospondin-1, which contribute to the proliferation of keratinocytes, ultimately accelerating wound epithelialization (Singh et al., 2022). If capillaries are obstructed or growth is arrested, ischemic ulcers can develop and transform into chronic wounds, as typified by occlusive atherosclerotic ulcers (Rodrigues et al., 2019). The repair phase lasts about 3 weeks. Finally, the maturation phase occurs, which can last up to 2 years. During this stage, scar tissue gradually replaces granulation tissue. As collagen increases, the tensile strength of the wound gradually increases, eventually reaching a peak (Almadani et al., 2021). It includes reactions such as epithelialization, wound contraction, and granulation tissue production (Jeschke et al., 2023) (Figure 1).

The most frequently occurring chronic wounds are diabetic foot ulcers (Armstrong et al., 2023), pressure sores, and venous leg ulcers (James et al., 2008). Chronic wounds are often accompanied by problems such as ischemia, necrosis, and infection (Hurlow and Bowler, 2022). Due to the long course of the disease, nursing measures and dressings become particularly important and bring a great burden to the patient’s daily life. The most commonly used wound dressing is gauze, which is widely applied in wound care and debridement procedures. Gauze is inexpensive and highly breathable; however, it has limited absorbency, poor biocompatibility, and lacks antibacterial properties. Consequently, various biomaterials have been developed for wound treatment (Boateng et al., 2008). Common wound dressings include electrospun fibers (He et al., 2024), hydrogels (Jiang et al., 2020) and microneedles (Yuan et al., 2022). Hydrogels, as biomaterial dressings that mimic the extracellular matrix, offer excellent biocompatibility, high absorbency, and good degradability, but they are prone to deformation, have insufficient mechanical strength, and cannot effectively facilitate transdermal drug delivery (Ho et al., 2022). Microneedles enable transdermal drug delivery and can achieve different therapeutic effects depending on the loaded drugs. However, they are complex to manufacture, have lower biocompatibility compared to hydrogels, and may cause minor wounds on the skin, which heal quickly after microneedle removal (Mo et al., 2023). Each of these wound dressings has its own advantages and limitations, and they can be combined to enhance wound treatment efficacy.

### 1.2 Hypoxia

Hypoxia plays a complex and critical role in wound healing. Wound healing is a multifaceted biological process that involves interactions among various cell types and molecules aimed at tissue repair, with oxygen being a crucial molecule that directly or indirectly affects multiple processes (Rafique et al., 2021; Konieczny et al., 2022). Under hypoxic conditions, oxygen concentration decreases in the wound area, stimulating the angiogenesis process (Huang F. et al., 2023). Angiogenesis is a critical step in wound healing, helping to restore blood supply to damaged tissue. Hypoxia activates various signaling pathways, including the induction of vascular endothelial growth factor (VEGF) expression, a key factor in promoting new blood vessel formation (Li et al., 2021). It has also been discovered that hypoxia stimulates fibroblast proliferation and migration, both essential for the formation of new tissue. Hypoxia can influence cellular metabolism in the wound area. Under hypoxic conditions, cells may shift from oxygen-dependent aerobic metabolism to anaerobic metabolism, which is less efficient but can sustain cell survival in low oxygen environments (Chun and Kim, 2021).

Short term tissue hypoxia after injury can induce wound healing. Hypoxia can upregulate vascular endothelial growth factors and the receptors, inducing new angiogenesis (Bao et al., 2009). It can also induce macrophages, keratinocytes, and fibroblasts to produce cytokines and growth factors. Long-term hypoxia, however, can hinder wound healing (Role of oxygen in wound healing, 2023). When the oxygen tension is too low, wound healing well be impeded, potentially leading to the transformation into chronic wounds (Tk and Hw, 1997). Currently, there have been many studies on improving tissue hypoxia to increase wound healing speed. For example, hydrogels that utilize reactive oxygen species (ROS) to achieve sustained oxygen release can increase oxygen levels in diabetic foot tissue, thereby promoting wound healing. (Guan et al., 2021; Yang et al., 2023). A polyvinyl alcohol/gelatin hybrid hydrogel loaded with oxygen-encapsulated exosomes has demonstrated enhanced angiogenesis and accelerated wound healing in a full-thickness wound model in male rats (Han et al., 2024). The role of hypoxia in wound healing is multifaceted, with both positive effects on healing promotion and potential negative impacts on healing obstacles. Precisely controlling oxygen concentrations in the local environment may optimize the wound healing process and accelerate tissue repair.

### 1.3 Vascular degeneration

Peripheral arterial disease (PAD) is a common circulatory system disorder characterized by arterial hardening, narrowing, or blockage in the lower limbs, usually resulting in reduced blood flow and inadequate blood supply to the legs (Criqui et al., 2021). In severe cases, it can lead to pain, ulcers, or even gangrene (Conte et al., 2019; Narula et al., 2018). PAD’s primary issue in wound healing is inadequate blood supply, as blood serves as the primary carrier of oxygen and nutrients. Consequently, wound healing processes in PAD patients are typically slow, with suboptimal outcomes (Prompers et al., 2008). In PAD, hypoxia in wounds and surrounding tissues due to poor blood circulation is common, hindering wound healing (Li B. et al., 2024). Poor circulation makes local tissues more susceptible to infection. Infections not only delay wound healing but also may exacerbate inflammation, further worsening local blood circulation in a vicious cycle (Juchniewicz and Lubkowska, 2020; Kohlman-Trigoboff, 2023; Aysert Yildiz et al., 2018). Due to inadequate leg blood supply, PAD often accompanies intermittent claudication or pain at rest, limiting patients' mobility. Reduced activity levels may further impair leg blood circulation, exacerbating wound healing issues. Therefore, wound management for PAD patients requires not only conventional wound care but also active improvement of local blood circulation through methods such as medication and physical therapy to promote wound healing.

### 1.4 Inflammation

Inflammation is the initial response following injury. The primary task of the inflammatory response is to clear the wound area of bacteria, viruses, dead cells and tissue debris through white blood cells, mainly neutrophils and macrophages. This provides a clean environment for subsequent cell proliferation and tissue rebuilding (Cooke, 2019). Throughout this process, the wound area emits various cytokines and chemotactic factors (Barrientos et al., 2008), which draw additional white blood cells and healing-associated cells, such as fibroblasts and endothelial cells, to the wound site, contributing to the subsequent healing process (Cooke, 2019). During the inflammatory phase, growth factors and cytokines not only recruit repair cells but also stimulate the formation of new blood vessels, a process called angiogenesis. The development of new blood vessels is vital for supplying oxygen and nutrients, as well as eliminating waste, all of which are essential for effective wound healing (DiPietro, 2016).

In the initial stage of wound healing, inflammatory reactions can activate immune and systemic defense processes. But long term inflammatory reactions will prevent wound healing, making wounds turn into chronic wounds (Eming et al., 2009). Multiple factors contribute to this phenomenon: Wound infection can lead to upregulation of inflammatory factors, delaying wound healing (Hurlow and Bowler, 2022). Inflammasome is one kind of multiprotein complex that is activated during infection or stress. It promotes the maturation of inflammatory factors (Schroder and Tschopp, 2010), pertaining to the development of pressure ulcers. In addition, abnormalities in macrophages and neutrophils also affect wound healing (Anderson et al., 2008). Inflammation is a critical component of wound healing, and managing the intensity and duration of the inflammatory response is vital for facilitating successful recovery. In clinical practice, appropriate anti-inflammatory treatments and measures to promote healing can help balance the inflammatory response and facilitate smooth wound healing.

### 1.5 Infections

Infection is one of the common reasons for delayed healing (Zheng et al., 2019). It can exacerbate and prolong the inflammatory response at the wound site. While inflammation is a necessary stage of wound healing, excessive inflammation can damage surrounding healthy tissue, produce more necrotic tissue, and hinder the healing process (Tang et al., 2020). Certain pathogens can produce toxins that directly damage cells and tissues, further disrupting the normal healing process of the wound (Rippon et al., 2022). A persistent infection can transform the wound into a chronic condition, which is difficult to heal and requires long-term management and treatment. Bacteria colonize the wound and form biofilm, which is an important factor that hinders wound healing and is difficult to remove (Sun et al., 2020). If a wound infection is not effectively managed, pathogens can disseminate to other areas of the body via the lymphatic system or bloodstream, resulting in more severe health complications, including sepsis (Li and Knetsch, 2018). Extended or inappropriate use of antibiotics for treating wound infections can lead to the development of antibiotic resistance in pathogens, complicating the treatment of the infection (Hurlow and Bowler, 2022). Infection can lead to the accumulation of more inflammatory cells and fibroblasts at the wound site, and excessive tissue repair responses may result in excess scar tissue formation, affecting both the function and aesthetics of the healed area (Antar et al., 2023). Common antibacterial strategies include the use of antimicrobial materials and antimicrobial drugs. Chitosan, known for its excellent antibacterial properties, carries positively charged groups on its surface that can disrupt bacterial cell structures, thereby achieving antibacterial effects (Jiang et al., 2020). Topical application of antibiotics can directly and rapidly treat infected wounds, but it faces challenges such as difficulties in transdermal drug delivery and short duration of drug release. Furthermore, some studies have explored the use of probiotic hydrogels to establish a beneficial microbial community on the wound surface, thereby reducing infection and inflammation (Ming et al., 2021).

One of the functions of the inflammatory stage is to reduce the infection of the wound (Guo and DiPietro, 2010). In clinical practice, the first steps in managing infected wounds involve controlling the infection, performing debridement, and creating a healing-friendly environment. Alongside the increased levels of inflammatory factors due to inflammation and the inhibitory effects on growth factors (Guo and DiPietro, 2010; Trøstrup et al., 2018), the biofilm produced by pathogenic bacteria can also contribute to delayed wound healing (James et al., 2008).

Cell scaffolds provide support, nutrition, and regulation for cell growth. The surface structure and modification groups of nanofiber scaffolds influence cell adhesion and proliferation (Wang et al., 2024). The chemical signaling between cells and the scaffold can affect the differentiation direction of cells, such as myocytes and osteocytes. The biocompatibility and cytotoxicity of the scaffold affect cell survival, while the porosity between fibers impacts the nutrient content, thereby influencing cell growth (Gasparotto et al., 2022). When the scaffold can release growth factors and induce angiogenesis, it further accelerates cell growth and tissue healing. An ideal scaffold should possess consistent mechanical support, resembling the extracellular matrix, as both overly soft and overly rigid scaffolds can impair cell growth (Youngblood et al., 2019). Additionally, it should have excellent biocompatibility, porosity, and controllable release capabilities for drugs and bioactive substances to meet the varying needs of tissues during different growth stages.

**FIGURE 2:**
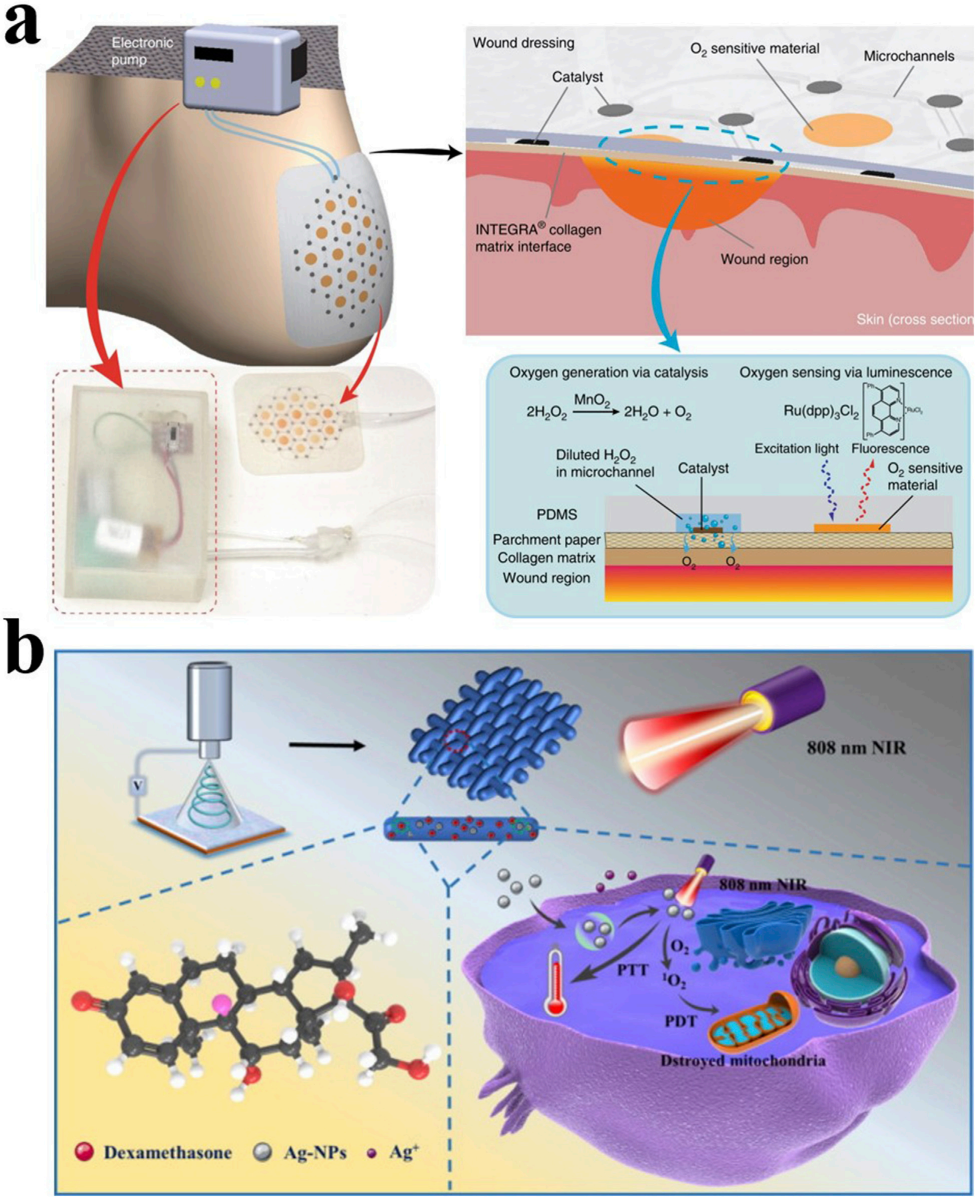
**(A)** Schematic diagram of a smart dressing for the treatment of foot ulcers that can increase the oxygen content of the wound (Ochoa et al., 2020). Copyright 2020, Microsystems & Nanoengineering. **(B)** Light-responsive PLLA/Dex/Ag fibers are used to treat bone infections (Liu et al., 2022). Copyright 2022, Materials & Design.

An ideal dressing should meet the following criteria: 1. Adjust the moisture of the wound environment and have a certain degree of breathability, 2. Mechanical stability against pressure and stress, 3. Promote cell migration and epithelialization, 4. Lower wound Adhesion, 5. Good elasticity and plasticity, 6. Complete barrier function to resist external adverse factors (Boateng et al., 2008; Nuutila and Eriksson, 2021) (Figure 2).

## 2 Electrospinning fundamentals

### 2.1 Principle of electrospinning

**FIGURE 3:**
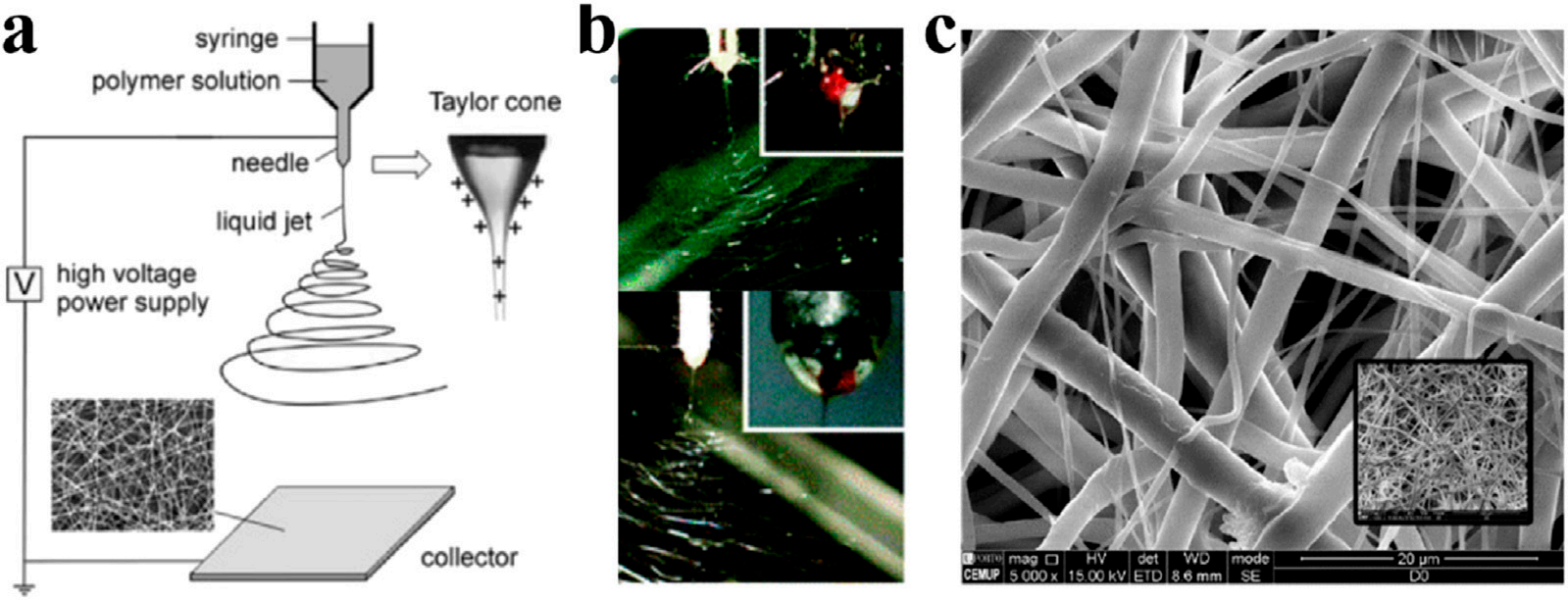
**(A)** One classical electrospinning setup consists of three parts: the high voltage power supply, the spinner and the collector. The charged solution is affected by electrostatic forces to form a jet, which ultimately forms nanofibers on the collector. Fiber morphology is affected by many factors, including electric field, solution type, nozzle structure and collector shape (Li and Xia, 2004a). Copyright 2004, Advanced Materials. **(B)** Side-by-side electrospinning using different spinnerets (Yu et al., 2017). Copyright 2017, Chemical Communications **(C)**. Electrospinning grid structure under scanning electron microscopy (Dias et al., 2022). Copyright 2022, Polymers.

A basic electrospinning setup consists of three parts: the components of electrospinning include a high-voltage power supply (which can be either direct current or alternating current), a spinner, and a collector. The high-voltage power supply charges the droplets, forming a jet that is stretched and shaped into fibers by the nozzle before being collected by the collector (Figure 3). Electrospun fibers have diameters ranging from 1 nm to several micrometers. In 1887, Charles V. Boys developed a device that facilitated electrospinning by connecting an insulated dish to an electrical power source (Xue et al., 2019). This technology underwent continuous improvement over the next century. By changing the raw materials, the voltage, the spinner or the collector, many different electrospinning fibers have been produced (Dong et al., 2022).

Conjugate electrospinning involves simultaneously electrospinning two different spinning solutions, resulting in nanofibers with oriented alignment deposited onto a collector to form a two-dimensional or three-dimensional composite fiber membrane. By combining polymers with different properties into the same composite membrane, the fiber membrane can acquire various functions (Xie et al., 2021).

Blended electrospinning refers to the process of mixing different polymers to prepare the spinning fluid, which is then electrospun using a single nozzle to obtain nanofibers with evenly distributed drugs. Alternatively, blended electrospinning can be achieved by using multiple nozzles simultaneously (Fattahi et al., 2017). This process is relatively simple and allows for the combination of different polymers or drugs based on specific needs. However, the drawback lies in the fact that it is simply a blending process, which cannot prevent interactions between different materials. This could result in drug precipitation from the polymer, leading to product instability. The property differences between the polymers may also cause structural instability and polymer separation (Christou et al., 2019).

Coaxial electrospinning is achieved by nesting capillary needles in the same direction, each equipped with different solutions to produce nanofibers with a core-shell structure. The outer nanofibers envelop the central core, which is why they are also referred to as core-shell nanofibers (Rajabifar et al., 2024). To prevent the solutions from merging before complete solidification, solutions with a low diffusion coefficient should be chosen. Core-shell electrospinning effectively addresses the incompatibility issues between polymers and drugs, and enhances drug loading capacity. The independent structure of the shell and core also provides better structural stability for these nanofibers (Dos Santos et al., 2022). Chitosan-polycaprolactone (CS-PCL) fibers feature a CS core embedded with tetracycline hydrochloride (TCH), with a smooth outer shell free of bead structures. The release profile of TCH from these fibers exhibits an initial burst release followed by long-term sustained release, ensuring prolonged antibacterial effects (Ghazalian et al., 2022). Co-electrospun poly (lactic acid)/gelatin nanofibrous membranes, crosslinked with glutaraldehyde, retain their nanofibrous and porous structures in the shell layer, which facilitates drug exposure and release (Rashedi et al., 2021).

Emulsion electrospinning utilizes an oil-in-water structure to emulsify bioactive agents, and then blends the emulsion with a polymer solution to produce nanofibers (Elyaderani et al., 2022). This method allows for the blending of polymers with different properties, such as hydrophilic and lipophilic compounds, without the need for organic solvents, thus reducing the generation of toxic substances. During the electrospinning process, the solution gradually dries and its viscosity increases, leading to differences in water content between the inner and outer layers. Hydrophilic compounds migrate inward, forming nanofibers with a core-shell structure (Norouzi et al., 2022). However, in some cases, the interfacial tension between solvents can damage the bioactivity of the loaded substances, imposing limitations on the choice of bioactive agents. Additionally, since oil-based polymers tend to accumulate on the outer side of the nanofibers, the product becomes more hydrophobic, which can impair cell adhesion and reduce biocompatibility (Mouro et al., 2023).

### 2.2 Classification by materials

In the early stage, electrospinning used polymers as raw materials, and then the types of materials gradually increased. Nowadays, in addition to traditional polymers (Huang et al., 2003), there are also several inorganic materials such as ceramics (Xing et al., 2021), carbon nanotubes (Wan et al., 2007), etc. Electrospun nanofibers have high porosity and a larger specific surface area, allowing them to form complex structures. Therefore, they can serve as scaffolds to support cell adhesion, promote angiogenesis, and achieve hemostasis and enhanced healing. However, the solvents used in electrospinning polymer fibers are difficult to recycle, potentially causing environmental pollution. Carbon nanotube fibers have high strength and structural stability, but they exhibit poor biocompatibility. Ceramic fibers face similar issues to carbon nanotube fibers. The structure of bioactive materials significantly impacts the performance of nanofibers. Increasing porosity can promote the infiltration of the extracellular matrix, while a rough surface favors cell adhesion and growth. Electrospun nanofibers made from natural materials offer better biocompatibility, but natural materials contain more impurities compared to polymers, are less stable in quality, and carry a higher risk of allergies. Surface modification groups on electrospun fibers influence cell growth, and while synthetic materials have high mechanical strength, their biocompatibility is poorer, leading to slightly inferior effects on cell adhesion and proliferation.

#### 2.2.1 Polymers

From 1964 to 1969, Jeffrey Taylor published a collection of papers that provided mathematical validation and modeling for the deformation of polymer solutions or melt droplets when subjected to strong electric fields (Geoffrey Ingram, 1964; Geoffrey Ingram, 1966). Polymer electrospinning can be classified into two categories based on raw materials: natural and synthetic polymers. These materials are extensively utilized across various fields, including biomedicine and tissue engineering (Chow, 2018). The raw materials of natural polymer electrospinning include collagen, fibrinogen, chitosan composites, alginate composites, gelatine composites, and silk fibroin (Wang F. et al., 2020). Natural polymers have high biocompatibility and low cytotoxicity, but they may contain more impurities during preparation, are difficult to maintain stable shapes, degrade quickly, and have poor spinnability. Synthetic polymers, on the other hand, offer high mechanical strength, stable and controllable products, and fewer impurities, but they exhibit poorer biocompatibility, and the synthesis process may generate harmful substances (Table 1). Natural and synthetic polymers can undergo structural modifications and derivatization to prepare semi-synthetic polymers, combining the advantages of both. However, issues such as polymer separation and insufficient solvent safety may still arise (Gutschmidt et al., 2021).

**TABLE 1:** Raw material classification.

Type		Material
Natural	Polysaccharides	Chitosan, Cellulose, Alginate, Hyaluronic Acid, Gelatin
	Protein	Silk Fibroin, Keratin, Collagen, Zein
Synthetic		Polylactic acid, Polybenzimidazole, Polycarboate, Polyurethanes, Polyethylene oxide

Blending them with synthetic polymers can improve this shortcoming. Such as blending synthetic polymers with SF (Zhang and Mo, 2013). Typical synthetic polymer electrospinning used in the medical field include polyglycolide (PGA), polyactide (PLA) (Zhong et al., 2023), poly (έ-caprolactone) (PCL) (Chen R. et al., 2023), polyurethane (PU) (Bhardwaj and Kundu, 2010).

Chitosan is a chitin derivative discovered in 1811 and was not used for wound treatment until the 1980s (Patrulea et al., 2015). It exhibited very low biological toxicity (Jiang et al., 2020). Dressings made with it have good hemostatic ability, biodegradability, and antibacterial ability (Wang W. et al., 2020; Chen et al., 2018). At the beginning of the 20th century, researchers began to extensively study the application of chitosan electrospinning in biomedicine. Chitosan electrospinning tissue engineering scaffolds, bone regeneration film, and wound dressings were developed at that time (Subramanian et al., 2005; Murugan and Ramakrishna, 2006; Zhou et al., 2008). Chitosan can be chemically modified to expand its range of applications. Carboxymethyl chitosan (CMC) is one of the most widely used derivatives. Compared with chitosan, it has better moisture retention and adsorption power, and the electrospinning performance of the prepared fiber is better (Sohofi et al., 2014). Quaternized chitosan is another important derivative that exhibits better antibacterial properties than chitosan and also has excellent biocompatibility and low toxicity (Cheah et al., 2019). In binary quaternized chitosan/chitosan fibers, the adhesion ability and degradation rate can be changed by adjusting the content of quaternizd chitosan (Andreica et al., 2023). Chitosan can work synergistically when combined with other substances. In 2013, researchers encapsulated silver nanoparticles in chitosan to create electrospun fiber mats, which showed antibacterial advantages (Abdelgawad et al., 2014).

Gelatin has excellent biodegradability and biocompatibility, and has natural physical and chemical properties similar to ECM. However, a simple aqueous gelatin solution easily gels at room temperature and is not suitable for preparation of electrospinning. Researchers have made a lot of efforts to increase its adaptability to electrospinning technology. (Li T. et al., 2022). Combining gelatin with other substances can enhance the mechanical strength and biochemical characteristics of the resulting product. Specifically, blending gelatin with PLLA improves the mechanical properties of the electrospun material, and these properties progressively strengthen as the content of PLLA increases (Yan et al., 2010). Electrospinning of gelatin and gelatin/poly (L-lactide) blend uses 2,2,2 trifluoroethanol (TFE) as the solvent, which has better ability to adjust the moisture of the wound surface. Not only can it moisturize, but it can also promote water discharge (Gu et al., 2009).

Silk is a material with a long history, and silk fibroin can be obtained after degumming. Unlike silk, silk fibroin has shown potential as a biomaterial. While it has good biocompatibility, it also has suitable mechanical properties, making it an ideal wound dressing (Çalamak et al., 2014). Initially, researchers concentrated on the impact of silk fibroin fibers on keratinocytes and fibroblasts. This type of electrospinning facilitates the adhesion of type I collagen and shows potential in dressings and tissue engineering scaffolds (Min et al., 2004). In addition, researchers have blended silk fibroin with other substances to develop wound dressings with antibacterial capabilities (Calamak et al., 2015). Cai et al. created a chitosan/silk fibroin electrospinning method. The addition of silk fibroin improved the mechanical properties of chitosan, while the increase in chitosan content will affect the fiber diameter, allowing for the production of finer electrospun fibers. This composite fiber also has certain antibacterial capabilities (Xiao et al., 2010).

Polycaprolactone (PCL) is a biodegradable aliphatic polyester. As a polymer, it has excellent structural stability with a slow degradation rate (Dias et al., 2022). These properties make it well suited as a carrier for long-term drug release. PCL/LAP/Ciprofloxacin e-spun nanofibers can sustain the release of drugs for more than 14 days (Kalwar et al., 2017). Zhang’s team achieved sequential release of drugs by encapsulating drugs in PCL and gelatin electrospun fibers respectively, and produced quad-axial nanofibers (Zhang et al., 2021). However, compared with natural substances, this type of electrospinning has poor biocompatibility and strong hydrophobicity. Table 2 shows several kinds of common polymer electrospinning and their application areas.

**TABLE 2:** Several common polymer raw materials. The product is widely used in industries, biomedicine, clothing, and other fields.

Polymers	Material	Solvents	Application	References
Polylactic acid	Synthetic	DOX/chloroform	Wound dressing/Drug delivery	Doustgani (2017)
Polybenzimidazole	Synthetic	-	Separation film for electrolyte film	
		Ethanolic KOH	Breathable fireproof inner garments production, for energy convertors components and as nanofibrous carbon precursor materials	Penchev et al. (2017)
Polycarboate	Synthetic	N,N-dimethylformamide (DMF), N,N-dimethylacetamide (DMAc), and dimethyl sulfoxide (DMSO)	Wound dressing/Drug delivery	Huang et al. (2022b)
Polyurethanes/boric acid	Synthetic	Dimethyl formamide	Protective textiles and highperformance filters	
Polyurethanes/CaCl2	Synthetic	Dimethyl formamide	Skin scaffold	
Polyethylene oxide/polycaprolactone	Synthetic	Chloroform	Drug delivery	Bani Mustafa et al. (2024)
Polystyrene	Synthetic	Chloroform	Textile industry	
Polyamide/chitosan	Hybrid	HOAc	-	Bochek et al. (2019)
Chitosan/HA	Natural	Acetic acid	Wound dressing	Sun et al. (2019)
Silk Fibroin	Natural		Scaffold/Wound dressing	Zong et al. (2018)
HA	Natural	Water	Wound dressing	Castro et al. (2021)

#### 2.2.2 Functional enhancement of electrospinning

In addition to polymers, electrospinning materials also include ceramics, carbon nanotubes (CNTs), and metals. CNTs are composed of one or multiple layers of graphene. This kind of material began to be widely studied in the 1990s (Iijima et al., 1992; Jishi et al., 1993). CNTs are stiff and strong. They have excellent electrical conductivity and great potential for development in composite materials. CNTs can be mixed with hydrogels, metals, and polymers to form composite materials (Wang et al., 2015; Cong et al., 2012). In 2003, SEOUL C and colleagues incorporated carbon nanotubes (CNTs) into a dimethylformamide (DMF) solution of poly (vinylidene fluoride) (PVDF) to prepare electrospun fibers, resulting in enhanced electrical conductivity and mechanical properties (Seoul et al., 2003). There are still some issues in the preparation of CNTs electrospinning that need to be resolved. The arrangement pattern of fibers is an important factor affecting the quality of CNTs electrospinning, and researchers have been committed to developing neatly arranged CNTs electrospinning (Sundaray et al., 2007). It has great application potential in tissue engineering, electronic sensors, cardiovascular, or consumer materials (Mejia et al., 2016; Chronakis, 2005; Zhang et al., 2022).

In the early 21st century, researchers began to introduce metals into electrospinning to prepare new composite materials (Watthanaarun et al., 2005). Electrospinning technology enables the straightforward production of pure nickel fibers, which exhibit distinct magnetic properties (Barakat et al., 2009). Metal electrospinning has great potential in energy and industry. Excellent conductive properties make it very suitable for the preparation of various sensors (Kailasa et al., 2021). In addition, electrospinning technology can also be used to absorb metal substances to purify water contaminated by heavy metals (Ma et al., 2011).

The theory of ceramic electrospinning technology is very mature, and there have been many related studies since this century. Ceramic nanofibers first rely on polymer formation conditions and then are calcined at high temperatures (Xing et al., 2021; Wu et al., 2012; Li and Xia, 2004b). Ceramic is inert and hard, and resistant to corrosion and chemical attack. In the early days of research, the material was largely confined to the laboratory. But recently it has been widely used in industry (Wang et al., 2022), environment (Malwal and Gopinath, 2016), chemical catalysis (Li et al., 2019) and biomedicine (Ferreira et al., 2021).

### 2.3 Preparation of electrospinning

The fundamental principle of electrospinning involves applying a strong electrostatic field to the polymer to create a Taylor cone at the capillary nozzle. When the electric field force surpasses the surface tension, a jet is formed, producing thin fibers that accumulate on the collector instead of forming droplets (Kim and Reneker, 1999; Bognitzki et al., 2001).

#### 2.3.1 Efforts to improve the productivity

As mentioned before, the traditional electrospinning device nozzle is a single needle. The production efficiency of nanofiber is limited. So the researchers improved the device and increased the output by increasing the number of deedles. However, this device will cause the jet to be blocked due to electric field interference when the needle distance is too close (30 mm) (Yang et al., 2010). The interference of electric field and the repulsion between the jets primarily depend on the spacing, quantity, and arrangement of the needles. Adjusting these parameters can enhance the electrospinning setup (Zhou et al., 2009a). In addition, stability can also be increased by introducing an auxiliary electrode to create a secondary electric field to neutralize the repulsion between the jets (Kim et al., 2006). Also, researchers have developed a single hole flat spinneret. The nanofibers produced by this device are similar in structure to those produced by traditional single-needle devices, at the same time have improved efficiency (Zhou et al., 2009b).However, the improvement of the nozzle did not solve the problems of low productivity and an unstable production process, so new production technologies were invented.

#### 2.3.2 Vibration electrospinning

Wang et al. summarized the vibration preparation process at that time, and applied it to electrospinning. The researchers applied vibrating force to the polymer, which significantly reduced its viscosity. The voltage required to produce electrospun fibers this way is reduced, and the products become finer (He et al., 2004). The addition of surfactants can further reduce the surface tension, thereby lowering the threshold voltage (Wang et al., 2008). Chen’s team developed the standing wave electrospinning in 2021. They added a self-created frequency modulator and oscillator, which reduces the threshold voltage to 18 kV through the additional inertial force generated by string vibration, thereby improving operational safety while producing finer electrospun fibers (Chen X. et al., 2021).

#### 2.3.3 Magneto-electrospinning

The technology of adding an external magnetic field outside the electrospinning device and using magnetic fluid as raw material is called magneto-electrospinning. In 2015, it was utilized to produce magnetic nanocomposite fibers containing α′′-Fe16N2 and α-Fe nanoparticles embedded in polyvinylpyrrolidone (Kartikowati et al., 2016). This technology provides better stability and finer products, but lacks practical applications and still needs further improvement. Tucker, BS et al. used PCL as the raw material, solubilized in 1,1,1,3,3,3-hexafluoro-2-propanol (HFIP), and achieved magnetization by placing a permanent magnet at the bottom of a quartz chamber. Compared to non-magnetized plasma, the oxygen/carbon ratio on the surface consistently increased. This approach permits the modification of the plasma plume shape for targeted functionalization of scaffold surfaces. Biomaterials underwent surface activation and functionalization via magneto-plasma processing (MPP) (Tucker et al., 2019). Magnetic poles added behind the concave current collector, the directionality of the generated nanofibers is increased, and the mechanical and thermal properties of the product are improved. When using this device to produce electrospinning, the output ratio of the products obtained at high temperatures is also higher than that of ordinary devices (Shehata and Abdelkader, 2018). An appropriate magnetic field can enhance the nanofiber orientation, improve mechanical and thermal properties, adjust the surface properties of nanofibers, and reduce the threshold voltage. These studies provide useful reference and inspiration for using magnetic field technology to improve the preparation and performance of electrospun nanofibers.

#### 2.3.4 Bubble electrospinning

**FIGURE 4:**
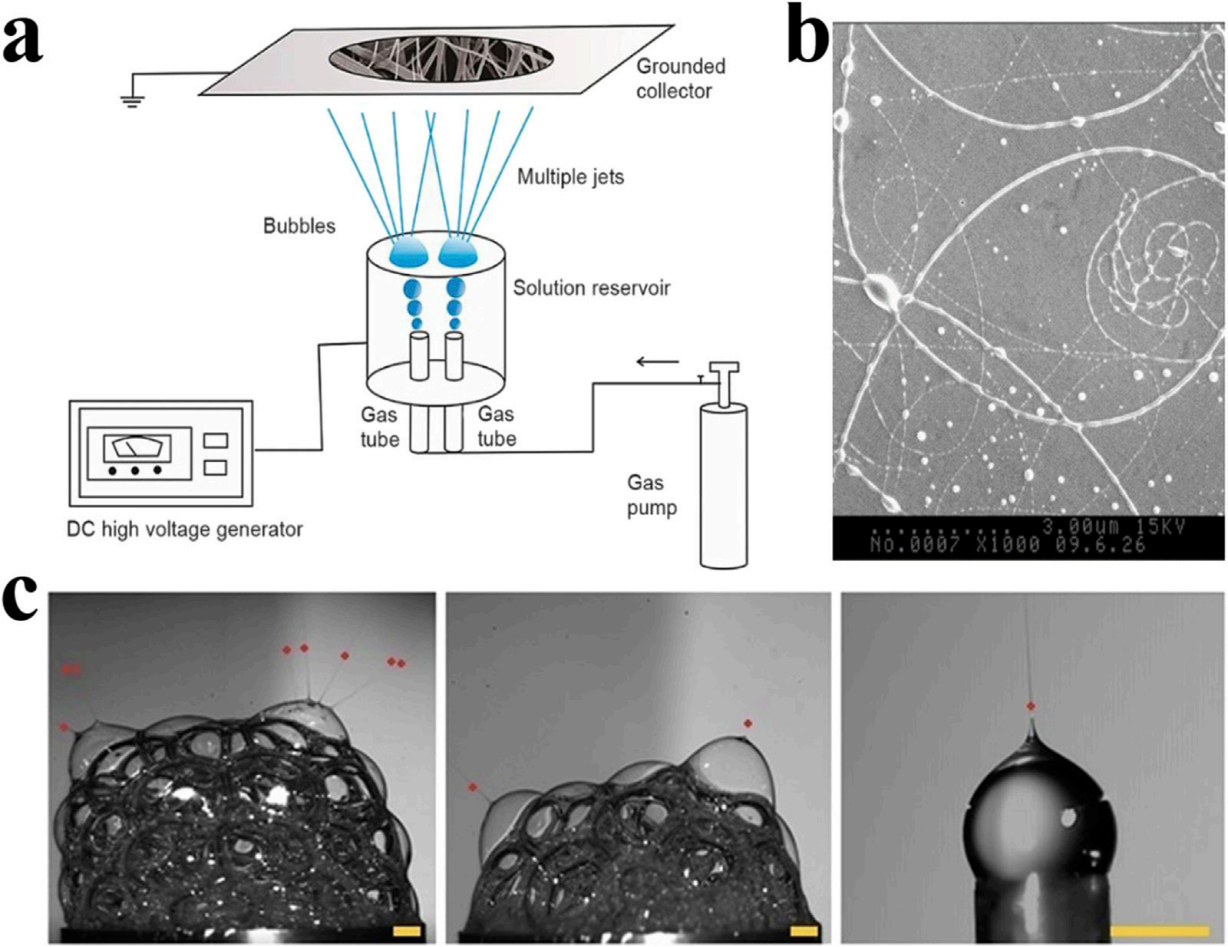
**(A)** The bubble electrospinning device forms multiple jets through the collapse of bubbles, thereby producing a large amount of electrospun fibers at the same time. However, these jets will interact with each other, resulting in changes in the product structure (Liu et al., 2023). Copyright 2023, Thermal Science. **(B)** Images captured by high-speed camera that show the Taylor cones and polymer jets (Ren and He, 2011). Copyright 2011, Journal of Applied Polymer Science. **(C)** SEM micrographs of electrospun fibers produced by bubble electrospinning (Erben et al., 2020). Copyright 2020, ACS Omega.

The technology of forming a bubble-induced cone through compressed air or nitrogen was proposed by Yong et al., in 2007, which was called bubble electrospinning (Liu and He, 2007). The main steps include bubble formation and control, bubble deformation, bubble rupture, and its fragments’ motion (He, 2020). Since multiple bubbles are easily formed on the surface of the solution and increase the number of jets, this technology is very suitable for mass production of electrospinning (Liu and He, 2007) (Figure 4). The technology uses an external force to overcome the surface tension of the bubble, causing it to break into millions of tiny jets, each of which can be picked up as nanofibers on a collector. This greatly increases production. But at the same time, the jets will interfere with each other in this production mode, and the morphology and mechanical structure of the fibers become difficult to control. Liu et al. proposed for the first time to predict the effects of two jets on velocity and fiber diameter, providing a direction to remedy this shortcoming (Liu et al., 2023).

### 2.4 Solid and hollow electrospinning

Most of the early electrospinning preparations were solid nanofibers (Shin et al., 2001). When nanofibers contain pores, their specific surface area increases dramatically (Katsogiannis et al., 2016). Techniques for increasing pore size in the fibers involve inducing phase separation while electrospinning and selectively extracting the sacrificial phase from the as-spun nanofibers through leaching or calcination methods (Xue et al., 2019). Porous MFe2O4 (M = Co, Ni, Cu, Mn) nanofiber produced by electrospinning has more exposed catalytically active site, resulting in improved electrocatalytic performance (Li et al., 2015).

Several methods exist for producing hollow electrospun fibers, including coaxial electrospinning, emulsion electrospinning, controlled heating of preformed nanofibers, and the application of a sacrificial template (Li et al., 2017). Dan Li et al. demonstrated that electrospinning using coaxial dual capillary nozzles can produce hollow nanofibers with a controllable hierarchical structure, and the double-layer surface of the fiber has independent and perfect functions. This process provides new ideas for producing catalysts with complex structures (Li et al., 2005).

### 2.5 Hydrogel-assisted electrospinning broadens nanostructure fabrication

Electrospinning has broad application prospects in tissue engineering and regenerative medicine due to its simple preparation, high yield and multifunctionality. However, in related research fields such as tissue engineering scaffold manufacturing, it is necessary to adjust the three-dimensional structure of electrospinning to better mimic the function of biological structures. Three-dimensional macroscopic nanostructures are flexible and varied, and the characteristic size can range from a few microns to a few millimeters. How to obtain appropriate spatial distribution and links between structures is one of the current research focuses. Seongsu Eom et al. proposed a novel hydrogel-assisted electrospinning process (GelES), using hydrogel as a collector to effectively improve the mechanical strength and permeability of the product. A three-dimensional mold with a cavity was made by three-dimensional printing technology, and the gelatin solution was injected into the cavity by utilizing the thermoreversible sol-gel transition of the hydrogel to obtain a hydrogel collector with a controllable three-dimensional structure. The electrospun nanofibers prepared by this method has both a hollow and drug-loaded structure, and has excellent mechanical strength and diffusion permeability, providing a new direction for the improvement of electrospinning technology (Eom et al., 2020).

### 2.6 Electrospinning device parameters

The main parameters influencing the product are: voltage, solution flow rate, distance between the needle and collector, solution viscosity, and solvent. The spinning voltage affects the diameter of the fibers. Higher voltage helps the solution overcome its own surface tension, causing the jet to stretch and split, resulting in smaller diameter fibers. However, when the electric field intensity is too high, the jet flow rate increases further during each spinning cycle, and the flow becomes too fast, which inhibits the stretching of the jet. This leads to reduced uniformity, increased diameter, and the fibers’ surface losing smoothness, resulting in bead-like or even beaded structures (Kalluri et al., 2021).

The solution flow rate is primarily determined by the speed of the pump. The flow rate influences both the production efficiency and the stability of droplet formation, as well as the diameter of the nanofibers. Unlike the effect of voltage, an increased flow rate leads to a larger diameter of the nanofibers. When the flow rate is too fast, the jet does not have enough time to fully dry and form before reaching the collector, resulting in bead-like or beaded structures (Bhattarai et al., 2018).

The distance between the needle and the collector affects the electric field intensity and the solvent evaporation rate. When other factors remain constant, a greater distance results in a weaker electric field. If the needle and the collector are positioned too closely, the solvent may not evaporate sufficiently, leading to irregular fiber surfaces or even fusion of the fibers (Sharma et al., 2023).

The solution viscosity influences the stretching properties of the polymer. If the viscosity is too low, the jet will break before reaching the collector, leading to the formation of bead-like structures. However, when the viscosity is too high, it obstructs the flow of the jet, causing the fiber surface to become irregular and resulting in structural defects. In such cases, an appropriate solution viscosity is essential for producing smooth, continuous fibers (Dodero et al., 2020).

The boiling point of the solvent affects its evaporation rate. A sufficient evaporation rate allows the jet to fully evaporate before being collected. However, if evaporation occurs too quickly, it can lead to needle clogging, hindering the formation of nanofibers. Additionally, most solvents used for polymer electrospinning are harmful to both the environment and human health, and their disposal can be challenging. This is an important factor to consider during the electrospinning process (Liu et al., 2015).

## 3 Electrospinning in Wound healing

Owing to their biocompatibility, surface area, and drug delivery capabilities, electrospinning has been increasingly used in wound treatment in recent years (Akhmetova and Heinz, 2020). Table 3 presents some electrospun dressings that have been applied in commerce. An ideal dressing should provide the best healing environment for the wound. It should have good air permeability, be able to regulate wound moisture, and have a low bacterial load (Boateng et al., 2008).The flexibility in structure and size makes electrospinning ideal for use as wound dressing (Wnek et al., 2003). Wound healing has very complex pH requirements. The skin naturally has an acidic environment, which can reduce the microbial load on the body surface. Maintaining an acidic environment in the wound can promote wound healing. However, when transplanting skin, an alkaline environment is required (Schneider et al., 2007). Therefore, dressings that can intelligently adjust wound pH have been under development. In 2021, Pakolpakcil developed a pH-sensing electrospun mat incorporating anthocyanins from purple cabbage (*Brassica oleracea*) for use as a wound dressing. The wound pH can be detected through the color change of the dressing (Pakolpakçıl et al., 2021).

**TABLE 3:** Examples of commercial electrospun products for wound healing.

Commercial products	Company and country	Stage of products	Electrospun material	Application
SurgiClot^®^	St Teresa Medical (United States)	Clinical use	Dextran	Hemostatic wound dressing
EktoTherix^®^	Neotherix, York, (United Kingdom)	Clinical Use	Polymer	Wound tissue engineering
Restrata^®^ Wound Matrix	Acera Surgical, Inc. (United States)	Clinical Use	Polyglactin 910 (PLGA 10:90) and polydioxanone (PDO)	Wound Healing
HealSmart™	PolyRemedy (United States)	Clinical Use	-	Wound Dressings
SpinCare™	Nanomedic Technologies (Israel)	Clinical Use	-	Commercialized electrospinning wound treatment portable device

### 3.1 Waterproof and breathable (W&B)

Benefiting from the porous structure and large surface area, the electrospun fiber pad has good breathability and moisturizing capabilities, which is very helpful in maintaining the wound environment. The performance of W&B aligned with the effects of the pore structure. When the maximum pore size of the fiber membrane is smaller than the diameter of a water droplet (>100 µm) and the minimum pore size is larger than that of a water vapor molecule (0.0004 µm), water vapor can pass through while water droplet infiltration is prevented. This allows EPU/FPU/Thymol nanofibrous membranes to exhibit excellent W&B performance (Yue et al., 2021). Additionly, self-pumping dressings have also been developed to treat wounds with excessive exudation. Lubin Zhou developed a self-pump dressing with the hydrophilic/hydrophobic double-layer structure. Cellulose non-woven fabrics are prepared into hydrophilic non-woven fabrics through alkalization, etherification, neutralization, washing, and drying. The PVP/PVB solution is modified with NaOH solution to make hydrophobic electrospun fibers. The hydrophilic modified non-woven fabrics absorb excess liquid, and the water channels on the surface of the hydrophobic electrospinning nanofibers further drain wound exudate. This type of dressing drains excess fluid while keeping the wound surface moist, which is beneficial for reducing the inflammatory response of the wound and promoting healing (Zhou et al., 2023). By embedding macromolecular antibacterial agents, this type of dressing can be endowed with antibacterial capabilities and further improve its efficacy (Qi et al., 2021).

### 3.2 Antibacterial function

Common raw materials used to prepare wound dressings include chitosan, silk fibroin, and polymers. Infection will slow down wound healing, so antibacterial ability is one of the important criteria for evaluating dressings. Adeli developed PVA/chitosan/starch nanofibrous mats and tested their anti-bacterial ability. They chose *Escherichia coli* and *Staphylococcus aureus* as experimental subjects. This electrospun mat showed obvious antibacterial ability and had a stronger inhibitory effect on *Staphylococcus aureus* than on *Escherichia coli*, possibly due to the difference of their cell wall structures (Adeli et al., 2019). Electrospinning can be used for drug encapsulation and sustained release, reducing the bacterial load in wounds over a long period of time. In 2014, Coskun developed electrospun poly (vinyl alcohol)/sodium alginate, which could adhere to the wound crust in the early stages of healing and demonstrated superior healing performance compared to other dressings. Researchers established a wound model on the skin of New Zealand rabbits to study the effects of nanofibers on wound healing at different time intervals. On postoperative day 21, the epidermis of wounds covered with nanofibers showed a more complete structure and well-formed appendages after healing. It can be thought of as artificial skin covering the surface of the wound before it heals (Coşkun et al., 2014). PVP combined with iodine forms a complex known as PVP-iodine (PVPI), which demonstrates exceptional antibacterial properties. Electrospun fibers made by this raw material show good air permeability, and their antibacterial capabilities can be easily improved by adding iodine (Dimatteo et al., 2018). In addition to the antibacterial ability of the fiber itself, electrospinnings can also achieve antibacterial purposes by releasing drugs. The fiber mats electrospun from a mixture of PVA, SF, ciprofloxacin, and epidermal growth factor exhibited the ability to regulate collagen and fibrin deposition, thereby modulating the extracellular matrix, promoting cell growth while also possessing antibacterial properties (Chouhan et al., 2017). Rath et al. synthesized gelatin nanofibers containing ZnO and cefazolin, which inhibited cell wall formation and disrupted bacterial structures to combat infection. ZnO demonstrated a reduction in cellular oxidative stress and alleviation of inflammation. Together, these components promoted wound healing (Rath et al., 2016; Mosallanezhad et al., 2022). Antibacterial substances such as ampicillin, indomethacin, and tannic acid can also be incorporated into nanofibers for sustained antibacterial effects (Bao et al., 2022; Norouzi and Abdouss, 2023). Although incorporating antibiotics is a simple and effective way to enhance antibacterial performance, interactions between the drugs and the fibers should be considered, as overuse of antibiotics can lead to bacterial resistance. Therefore, antimicrobial strategies based on the unique properties of polymers should be a focus of future research. For example, Chen et al. utilized the antibacterial properties of silver (Ag) combined with the anti-inflammatory capabilities of curcumin to create nanofibers from gelatin and chitosan, which exhibited antibacterial, anti-inflammatory, hemostatic, and healing-promoting effects (Chen K. et al., 2021).

### 3.3 Drug delivery

The large surface area and structural flexibility of electrospinning make it ideal for drug delivery. Electrospinning can load a variety of drugs, including antibiotics, anti-cancer agents, proteins, and DNA. Electrospinning for drug delivery should be evaluated for drug loading, release capacity, degradability, and biocompatibility. Jing Z encapsulates various surface active substances and drugs into electrospun fibers prepared from PLLA solution to make fiber mats containing drugs. Rifampicin drugs were observed to be evenly distributed within the fibers and released through the degradation of PLLA fibers (Zeng et al., 2003). Ji S relied on the excellent conductive properties of CNTs to prepare an electrosensitive transdermal drug delivery system made of polyethylene oxide and pentaerythritol triacrylate polymers. It can increase drug release as the voltage increases and does not affect the activity of mouse fibroblasts (Im et al., 2010). Research has shown that pH affects drug loading and release, suggesting that drugs can be bound to electrospun fibers and triggered to release in response to pH changes (Jassal et al., 2015). In 2022, Poly (vinyl alcohol) (PVA)-graphene oxide (GO)-silver (Ag) nanofibers containing curcumin (CUR) were developed as wound dressings. This fiber has pH responsiveness. When the pH rises, CUR will stop releasing, while the acidic environment will promote the release of CUR to achieve a dynamic adjustment effect. Due to the synergistic effect of GO-Ag and CUR, this fiber exhibits excellent antibacterial properties. It also has positive effects on the migration and proliferation of NIH 3T3 fibroblasts (Rahmani et al., 2022).

By adjusting the degradation rate of the polymer, the delivery time of the drug can be further controlled to achieve long-term release. Kielholz T’s team electrospun drug-loaded PVP fibers and pure Eudragit E fibers in parallel for the release of antimicrobial peptides. They compared the *in vitro* drug release curves of parallel electrospun and pure PVP fibers within 24 h. Pure PVP fibers released all the drugs after 30 min, while incorporating 20% Eudragit E could reduce the drug released at 30 min to 73%, and the delay effect increases with the proportion of Eudragit E. When the content of Eudragit E is enough, the sustained release time of the drug can reach more than 24 h. The effect of fiber mats on cell viability was tested with human keratinocytes (HaCaT, kindly provided by Prof. N. Fusenig, German Cancer Research Center, Heidelberg, Germany) with a lactate dehydrogenase (LDH) assay, When the drug concentration is too high, the biological activity of the cells may be reduced. This issue should be taken into account when selecting the formulation (Kielholz et al., 2022). Mehraz et al. developed silk fibroin/β-cyclodextrin citrate nanofibers in 2020. They used a solution of covalently linked silk fibroin and cyclodextrin to prepare electrospun fibers, loaded with rotated ciprofloxacin, and stably released the drug. The time can reach about 50 h. And it is possible to control the total amount of drug release-cyclodextrin ratio through polymer modification (Mehraz et al., 2020).

**FIGURE 5:**
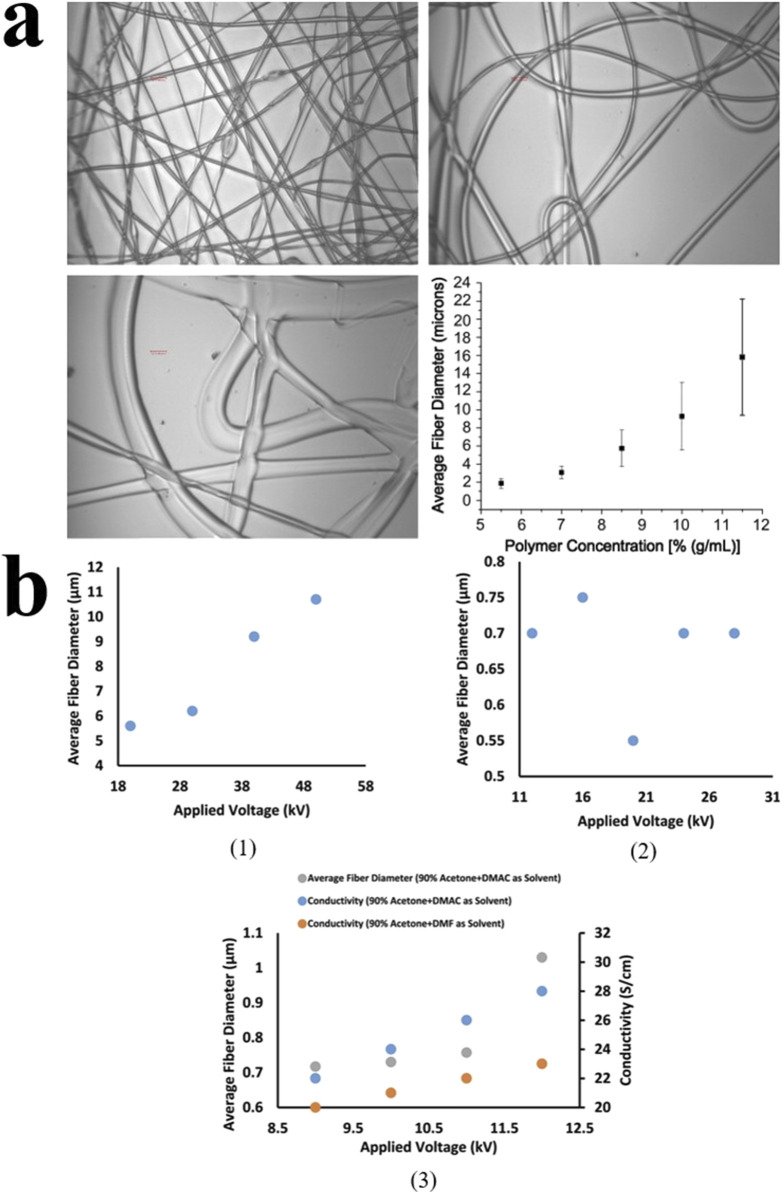
**(A)** The effect of polymer concentration on nanofiber diameter under electron microscopy. When the concentration exceeds 11.5%, insufficient drying leads to fiber adhesion. (Copyright 2008. Elsevier) **(B1)** The effect of voltage on the average fiber diameter of the solution. **(B2)** The effect of solvent concentration on the average fiber diameter. **(B3)** The effect of solvent type on conductivity and average fiber diameter (Copyright 2023. Elsevier).

**FIGURE 6:**
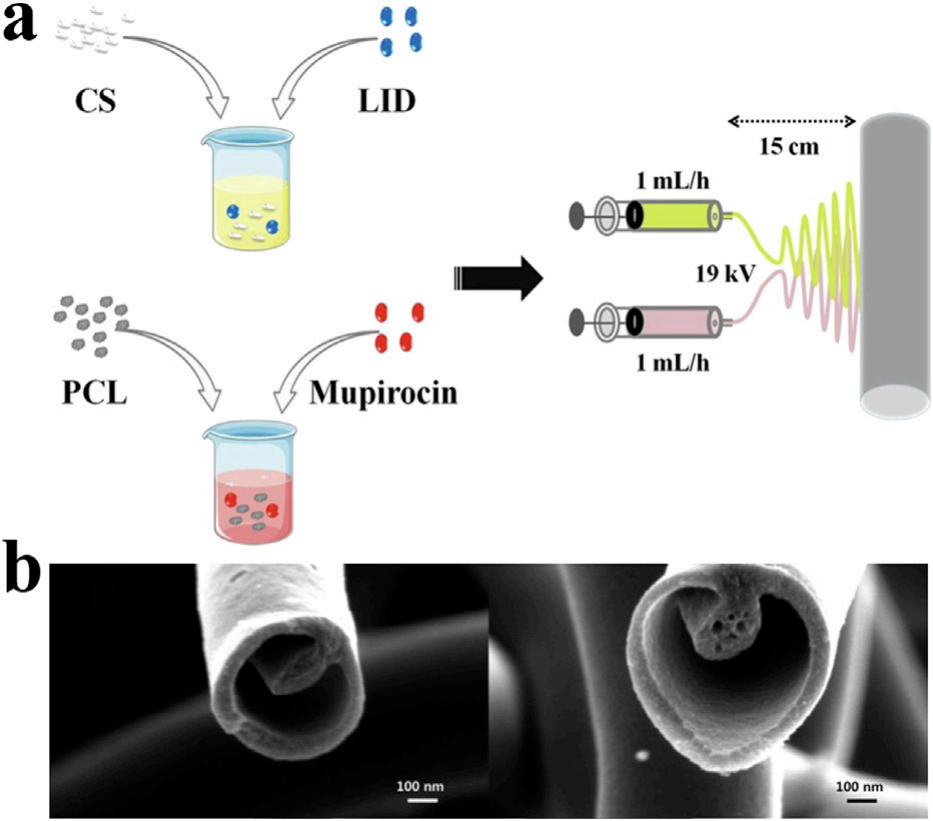
**(A)** The dual spinneret electrospinning apparatus assembly which producing lidocaine hydrochloride (LID) and mupirocin-loaded chitosan/polycaprolactone (CSLD-PCLM) scaffolds (Yang et al., 2020). Copyright 2020, ACS Biomaterials. **(B)** Tri-layered nanofibers produced with a tri-layered core-cut nozzle (Lee et al., 2014). Copyright 2014, Scientific Reports.

In addition to differences in composition, different electrospinning structures also affect the period of drug release. Haixia Xu’s team prepared a layered film as a wound dressing. The top layer is PCL, loaded with zinc oxide nanoparticles (n-ZnO). The middle layer is prepared by parallel electrospinning technology to prepare Janus fibers, and loaded with n-ZnO and ciprofloxacin (CIP) at the same time. The bottom layer is gelatin, loaded with CIP. The top layer of PCL relies on its good hydrophobicity and the antibacterial properties of n-ZnO to protect the wound surface and block external pollution. The underlying gelatin has good biocompatibility and allows the drug to directly contact the wound surface, providing rapid release of antibacterial drugs. The middle layer promotes drug release and acts as a scaffold to ensure structural continuity. This dressing can ensure stable drug release within 24 h (Xu et al., 2022). In the application of certain cytotoxic drugs, a reasonable structure can optimize the release mode, thereby reducing toxic side effects and improving therapeutic effects. Sanaz Alizadeh’s team prepared core-shell micro/nanofibers through emulsion electrospinning. The shell is made of SSA and PVA, and the core is made of PCL. The SSA on the shell is negatively charged and captures copper ions. The PVA optimizes the mechanical properties of the shell and increases storage capacity. The PCL serves as the core structure to load CuO NPs of different concentrations to provide sustained and controllable CuO release. Experiments show that the cytotoxicity of CuO NPs is concentration-dependent. This core-chell micro/nanofibers can load CuO NPs at a concentration of 8% w/w and sustainably release NPs in smaller amounts (Alizadeh et al., 2024). Another similar studies is Li A’s core/shell structure dressing prepared by coaxial electrospinning technology, loaded with Epigallocatechin-3-O-gallate (EGCG), which shows sustained drug release capabilities, excellent biocompatibility, and achieves precise drug delivery (Li A. et al., 2022) (Figures 5, 6).

### 3.4 Hydrogel nanofiber fabricated by electrospinning technology

As a highly biocompatible and degradable cell matrix material, hydrogel has been widely studied in wound treatment. It is easy to modify and can obtain products with multiple functions (Habib et al., 2015). Hydrogels are often lacking in mechanical strength. Researchers combine hydrogels with electrospinning to enhance the functions of both. Xiao He et al. used electrospinning technology to prepare hydrogel nanofibers containing citric acid (CA), acrylic acid (AA) and polyacrylamide-co-acrylate (P (AAm-co-AA)). This fiber has long-lasting adhesion, strong antibacterial activity and strong spinnability. This property makes it very suitable for the preparation of masks and wound dressings. However, the healing ability of this nanofiber for wounds remains to be explored (He et al., 2021).

Chitosan (CS, C6H11NO4)n is a product of chitin N-deacetylation, which can dissolve in inorganic acids such as hydrochloric acid to prepare CS solutions (Chen et al., 2018). The dissolved CS carries a positive charge, exhibiting excellent antibacterial properties and water absorption capabilities. It has been widely applied in the biomedical field, such as for the fabrication of tissue engineering scaffolds, wound dressings, and drug delivery systems. Nanofibers made from CS possess excellent biocompatibility and antibacterial properties, but they lack mechanical strength, making it difficult to maintain the stability of surrounding tissue morphology (Bochek et al. 2019). This issue can be addressed by incorporating cellulose nanofibers (CNF) as a structural framework. In contrast, electrospun fibers made from CS as the primary material exhibit better tensile and compressive properties. Improving mechanical strength while maintaining the advantages of CS is a key challenge that needs to be addressed.

Dangwei Li constructed a CS/cellulose nanofiber/tannic acid (CS/CNF/TA) antibacterial hydrogel. The researchers assembled CS, CNF and TA into a hydrogel network with a three-dimensional structure by forming hydrophobic bonds and hydrogen bonds among them. CNFs prepared by electrospinning were incorporated into the hydrogel as scaffolds, significantly improving its mechanical strength. Uniformly distributed three-dimensional pores and microporous fiber distribution can be seen under SEM. The researchers added tannic acid to form hydrogen bonds and electrostatic interactions between TA and CS/CNF to maintain structural stability under large pores. The surface of the CS/CNF/TA hydrogel network carries positive charges and can react with bacteria with negative charges on the surface to achieve a bactericidal effect. In the tissue defect model, the hydrogel can absorb wound exudation while keeping the wound moist. Combined with its antibacterial effect, it provides a moist and sterile environment to promote wound healing. An experimental group established a skin defect model on the body surface of male rats and treated the wounds with CS/CNF/TA hydrogel. The healing speed and ratio of the wounds in this treatment group were significantly higher than those of the control group. Additionally, in mice infected with *Staphylococcus aureus*, the healing speed was slower than that of the blank control group, which further demonstrates the importance of antibacterial function in wound healing (Li D. et al., 2024).

In addition to directly spinning hydrogels, electrospinning can also be combined with hydrogels to improve the properties of both. Pebax solution, a resin with excellent thermoplastic and tensile properties, has been widely used in fields such as food packaging and medical devices. Hajar Rajati developed a polyamide/Pistacia atlantica (P.a) gum nanofiber fabricated by electrospinning technology. SEM analysis showed that the product was an extremely fine continuous fiber with an average diameter below 200 nm. They prepared the Poly (etherblock-amide) (PEBAX)/PVA/Ag hydrogel solution casting method, and coated the electrospun nanofiber on it. Compared with untreated hydrogels, after adding the electrospun nanofiber layer, the product’s water absorption capacity, air permeability and degradation ability have been improved, and the tensile strength has also been greatly improved. In addition, through *in vitro* fibroblast survival assays in mice, it was concluded that this type of hydrogel exhibits no cytotoxicity, making it suitable for wound treatment (Rajati et al., 2023).

### 3.5 Prospects and challenges

Electrospinning technology is simple to implement, high in yield, and capable of providing multifunctional, sustained drug delivery. As a non-invasive treatment, it shows great potential in the treatment of chronic wounds. Relevant cell and animal experiments have been thoroughly developed, and its biocompatibility, water absorption capacity, and ability to promote cell adhesion, proliferation, and angiogenesis have been well-documented. However, its widespread application still faces numerous challenges. Comparative studies of different electrospinning techniques are relatively scarce, which makes it difficult to select the optimal technological solution. The biocompatibility and mechanical properties of electrospun materials still need further optimization. While research has shown that material modification can enhance the mechanical strength and biocompatibility of fibers, balancing the mechanical properties with biodegradability remains a significant challenge in practical applications. Future research could explore new composite materials or further improve material properties through surface modification techniques.

Another important challenge is the production efficiency and cost of electrospinning. Although electrospinning technology offers high flexibility, some raw materials and solvents may cause environmental pollution. Future studies should focus on developing more efficient and environmentally friendly electrospinning processes to reduce production costs and minimize environmental impact. Another key issue is how to precisely control the drug release rate and dosage, as well as how to prevent drug inactivation during the release process. The most commonly used clinical dressings are sterile gauze, but compared to gauze, electrospun materials offer superior antibacterial properties, biocompatibility, water absorption, and drug release capabilities. Although the manufacturing cost is higher than that of gauze, electrospun materials can reduce adverse events in wound treatment and shorten healing time, making them highly promising and valuable for future applications.

## 4 Conclusion

Wounds result from skin damage and can progress to chronic wounds if left untreated, leading to substantial healthcare costs. Surgical incisions and diabetic foot ulcers are among the costliest wound types, impacting a large number of individuals each year. Preventing wound infections, providing proper care, and utilizing suitable dressings are crucial in the wound healing process.

Electrospinning, a technique with roots in the early 21st century, has garnered attention for its potential in wound treatment. This technique generates nanofibers by applying an electric field, providing several benefits for wound care applications. The ability to tailor fiber orientation and alignment makes electrospun nanofibers ideal for promoting tissue regeneration and wound healing.

Recent researches have investigated the application of electrospun nanofibers in wound healing, demonstrating their efficacy in promoting cell growth and tissue regeneration. Looking ahead, the future of electrospinning in wound treatment appears promising. Continued research efforts aimed at optimizing material selection, fiber design, and fabrication techniques will further enhance the efficacy of electrospun nanofibers for chronic wound management. Advancements in functionalizing electrospun materials, such as incorporating bioactive agents and growth factors, hold potential for accelerating wound healing processes and improving patient outcomes. Core-shell electrospinning, as a widely studied classic structure, holds great potential for applications in drug release, wound healing, and tissue scaffolding. Hybrid fibrous membranes (HFMs) compensate for the respective shortcomings of hydrogels and electrospun fibers, thereby enhancing therapeutic efficacy. Although some electrospun dressings have already been approved for clinical use, most research products have not yet received FDA approval. The majority of the relevant research remains confined to laboratory environments and has not been applied in clinical settings, lacking clinical data. Additionally, there is a lack of comparison between different electrospinning techniques, making it difficult to make horizontal comparisons. It is hoped that future research can address this deficit.
